# Camera-based Prospective Motion Correction in Paediatric Epilepsy Patients Enables EEG-fMRI Localization Even in High-motion States

**DOI:** 10.1007/s10548-023-00945-0

**Published:** 2023-03-20

**Authors:** Mirja Steinbrenner, Amy McDowell, Maria Centeno, Friederike Moeller, Suejen Perani, Sara Lorio, Danilo Maziero, David W. Carmichael

**Affiliations:** 1 School of Biomedical Engineering and Imaging Sciences, King’s College London, St. Thomas’ Hospital, Westminster Bridge Road, London, SE1 7EH UK; 2 Developmental Imaging and Biophysics, UCL Institute of Child Health, University College London, 30 Guilford St, London, WC1N 1EH UK; 3 Department of Radiation Medicine & Applied Sciences, University of California, San Diego Health, San Diego, CA USA; 4 Department of Clinical Neurophysiology, Great Ormond Street Hospital, Great Ormond Street, London, WC1N 3JH UK; 5 Department of Basic and Clinical Neuroscience, KCL Institute of Psychiatry, Psychology & Neuroscience, 16 De Crespigny Park, London, SE5 8AF UK; 6 Department of Neurology and Experimental Neurology, Epilepsy Center Berlin-Brandenburg, Charité–Universitätsmedizin Berlin, Hindenburgdamm 30, 12203 Berlin, Germany; 7 Epilepsy Unit, Neurology Department, Hospital Clinic Barcelona/IDIBAPS, Villarroel 170., Barcelona, 08036 Spain

**Keywords:** Prospective motion correction, EEG-fMRI, Drug-resistant epilepsy, Pediatric epilepsy

## Abstract

Background: EEG-fMRI is a useful additional test to localize the epileptogenic zone (EZ) particularly in MRI negative cases. However subject motion presents a particular challenge owing to its large effects on both MRI and EEG signal. Traditionally it is assumed that prospective motion correction (PMC) of fMRI precludes EEG artifact correction. Methods: Children undergoing presurgical assessment at Great Ormond Street Hospital were included into the study. PMC of fMRI was done using a commercial system with a Moiré Phase Tracking marker and MR-compatible camera. For retrospective EEG correction both a standard and a motion educated EEG artefact correction (REEGMAS) were compared to each other. Results: Ten children underwent simultaneous EEG-fMRI. Overall head movement was high (mean RMS velocity < 1.5 mm/s) and showed high inter- and intra-individual variability. Comparing motion measured by the PMC camera and the (uncorrected residual) motion detected by realignment of fMRI images, there was a five-fold reduction in motion from its prospective correction. Retrospective EEG correction using both standard approaches and REEGMAS allowed the visualization and identification of physiological noise and epileptiform discharges. Seven of 10 children had significant maps, which were concordant with the clinical EZ hypothesis in 6 of these 7. Conclusion: To our knowledge this is the first application of camera-based PMC for MRI in a pediatric clinical setting. Despite large amount of movement PMC in combination with retrospective EEG correction recovered data and obtained clinically meaningful results during high levels of subject motion. Practical limitations may currently limit the widespread use of this technology.

**Supplementary Information:**

The online version contains supplementary material available at 10.1007/s10548-023-00945-0.

## Introduction

### Potential of EEG-fMRI

Simultaneous EEG-fMRI was developed to try to map the generators of epileptic discharges (Lemieux et al. 2001) increasing the localization accuracy compared to EEG alone. In addition to its potential relevance as a tool in the presurgical evaluation of pediatric epilepsy (Centeno et al. 2017), it has been a key tool in the development of our pathophysiological understanding of epilepsy (Centeno and Carmichael 2014).

Focal epileptic discharges are often associated with widespread activity that is indexed by Blood Oxygenation Level Dependent (BOLD) fMRI signal changes leading to a greater understanding of the networks involved in temporal lobe epilepsy (TLE) (Laufs et al. 2014), extra TLE (Centeno et al. 2017) and generalized epilepsy (Aghakhani et al. 2004; Moeller et al. 2010).

While the majority of studies have examined interictal epileptic networks, it is also possible to non-invasively localize the seizure onset zone by mapping preictal and ictal hemodynamic changes, which maybe especially useful in patients where information from ictal scalp-EEG is not sufficient for localization (Chaudhary et al. 2012).

### One Major Limitation in General but Particularly for Pediatric Cohorts is Subject Motion

Subject motion of only a few millimeters during the acquisition of EEG-fMRI can degrade fMRI data quality substantially (Hajnal et al. 1994; Satterthwaite et al. 2012). One way to approach this problem is to correct for motion in fMRI retrospectively, though in several approaches like scan nulling or scrubbing there can be a relevant data loss (Lemieux et al. 2007; Power et al. 2014). FIACH (Functional Image Artefact Correction Heuristic) is a tool which has been shown to effectively remove non-physiological signal changes in a two-step procedure by identifying and correcting large amplitude signal changes and modelling the effects of regions of high temporal instability (Tierney et al. 2016b). While a variety of other post-processing methods can be used to reduce the impact of motion, data during unique clinical events cannot be discarded and residual errors can make results hard to interpret (Power et al. 2015) or even lead to false activations (Hajnal et al. 1994).

### EEG Data Quality Corruption

There are two major contributors to head motion-related degradation of EEG quality acquired with simultaneous fMRI. The first is related to the Faraday’s law of induction, where the temporal derivative of the magnetic flux through an area composed by electrode, wire and head may induce voltages that will be added to electrical signals measured by the electrode. The second motion-related artefact is associated with the disruption of the gradient artefact’s temporal stability. Typically, the gradient artefact (GA) correction is performed by averaging consecutive EEG epochs to create a template and further subtracting it from the total signal measured. This operation considers that the voltages generated by the applied time varying magnetic fields to acquire fMRI data are similar for successive fMRI slices or volumes (Allen et al. 2000). Therefore, GA correction becomes more challenging when motion decreases its stationarity. However, recent work (Maziero et al. 2016) suggests that though the temporal stability of the GA is reduced by motion, it is not further reduced by applying effective prospective motion correction (PMC) during fMRI data acquisition. In fact, applying PMC to fMRI data seeks to maintain the spatial relationship between the EEG equipment (moving with the head) and the switched magnetic fields used for imaging in terms of their relative angle. The motion information can be used to model and remove GA temporal changes (Maziero et al. 2021) and vice-versa (Laustsen et al. 2021).

### Applications of PMC

Previous work in analyzing the use of PMC has mainly focused on an experimental setting with healthy subjects and a motion, no-motion condition (Callaghan et al. 2015; Todd et al. 2015; Zaitsev et al. 2006; Maziero et al. 2020). Here, for the first time we use prospective motion correction in a pediatric clinical population undergoing EEG-fMRI using a commercially available motion correction system.

The application of PMC in a pediatric clinical cohort has the possible advantage of bypassing the need for sedation, which reduces motion, but potentially suppresses epileptic activity and thereby lessens the diagnostic yield (Siniatchkin et al. 2018). Image realignment is an effective strategy to reduce motion effects (Friston et al. 1996) however it can only correct motion occurring on a timescale substantially less than the rate of image acquisition (typically several seconds). This leaves residual motion that can cause large amplitude residual signal changes (Tierney et al. 2016b; Beall and Lowe 2014). One option is to effectively remove affected volumes using additional regressors within the general linear model (Lemieux et al. 2007), however, this affects detection power particularly when many volumes are affected. Prospective motion correction can mitigate these effects by updating the acquisition of each slice (at a timescale of 50-100ms) to remain consistent with the subject’s anatomy by updating imaging gradients. However, it relies on accurate knowledge of subject’s position to correctly move the imaging slice, otherwise it can potentially introduce unwanted variability in slice location (for example when the subject is still) leading to greater fMRI signal instability. EEG artefact correction relies on the temporal repetition of the artefact created by switching magnetic field gradients for imaging. In previous work, we have shown that provided the motion information is accurate then the EEG gradient artefact is sufficiently stable, although this is reliant on maintaining the same relative orientation between the imaging gradients and EEG circuitry (Maziero et al. 2020). Therefore, reliable prospective motion correction has the potential to reduce motion-related noise in fMRI while maintaining EEG quality. However, inaccurate motion information will lead to corruption of both EEG and fMRI during prospective motion correction.

The goal of the current study was to perform EEG-fMRI with prospective fMRI correction and retrospective EEG correction and evaluate the feasibility and effectiveness of this approach. In particular we wanted to:

1. Assess EEG quality during prospective fMRI correction using standard and motion model EEG correction approaches.
2. Assess if effective suppression of head motion in fMRI could be achieved in a clinical pediatric population.
3. Use these to evaluate the motion tracking performance.
4. Provide face validity of the epilepsy localization achieved using motion correction.

## Materials and Methods

### Subjects

Children (4 Female, mean age: 14 Years), with drug Resistant Focal Epilepsy Underwent Simultaneous EEG-fMRI.

Recruited patients were undergoing evaluation for epilepsy surgery at Great Ormond Street Hospital (London, UK). Inclusion criteria for the study were: participant ages between 8 and 18, diagnosis of drug-resistant focal epilepsy and frequent epileptiform discharges in their video-EEG telemetry.

### Motion Measurement and fMRI-prospective Motion Correction

Motion parameters were continuously recorded throughout the whole scan as has been described in previous work coming from our group (Maziero et al. 2021): In summary a MR-compatible camera (Metria Innovation Inc., Milwaukee, USA) was used for tracking a Moiré-Phase-Tracker (MPT) marker (Maclaren et al. 2013) attached to a ‘bite bar’ that was specifically produced for each subject based on a dental retainer. The camera-tracker system was used to record head motion with six degrees of freedom, three translations (x, y and z axis, defined as right–left, posterior–anterior and feet–head, respectively) and three rotations (about x, y and z axis, respectively) with a sampling rate of 80 Hz. The motion parameters were logged on a computer located outside the scanner room. The motion parameters were used to update the Radio Frequency (frequency offset encoding slice position) and Magnetic Field Gradient orientation before the acquisition of every fMRI slice (Maclaren et al. 2013; Todd et al. 2015) to achieve prospective motion correction of fMRI data.

### EEG-fMRI Acquisition

Patients were prepared with a 64-electrode MRI-compatible EEG cap (Easy Cap; Brain Products, Gilching, Germany). EEG data was acquired using a sampling rate of 5 kHz, band-pass filtered at 0.016 Hz-1 kHz with 16-bit digitalization (0.5µV resolution). Prior to starting the simultaneous EEG-fMRI, a ten-minute duration baseline EEG recording was acquired.

All images were acquired at Great Ormond Street Hospital, London, United Kingdom, using a 3T MRI System (Siemens Prisma, Erlangen, Germany) with a 64-channel head-neck RF coil.

A T1 weighted MP-RAGE structural image was obtained (1mm isotropic resolution TE/TR 2.74/2300ms, FA = 8 deg). Two to three 12 min 3D- echo-planar imaging (3D-EPI) sessions were acquired (TE/TR 28/2400 ms, 2.5 × 2.5 × 2.5 (plus 0.5 gap), 40 sequential axial slices, field of view (FOV) 200 × 200 mm^2^, 80 × 80 matrix).

### EEG Post-processing

We used two different approaches to correct artefacts present on the in-scanner EEG data. Firstly, Average Artefact Subtraction (AAS), as implemented within BrainVision Analyzer2 (Brain Products, Germany) was used to remove MR gradient (Allen et al. 2000) and pulse-related artefacts from the EEG (Allen et al. 2000). The EEG was then down sampled to 250 Hz and filtered between 0.5 and 70 Hz for visual review.

Secondly, we applied REEGMAS (Maziero et al. 2016), an in-house code developed in Matlab 2015a (www.mathsworks.com). REEGMAS consists of a linear regression of a model derived from the motion parameters that is applied to the EEG data before proceeding with standard gradient artifact (GA) and cardiobalistic artifact (BCG) correction by AAS (Maziero et al. 2021). We averaged 15 slices, each with a slice TR = 60ms, to generate a gradient artefact template for both BrainVision and REEGMAS. To apply REEGMAS, a model of motion induced voltages is formed from the 6 motion parameters (3 translation and 3 rotations) recorded by the MPT-camera system, their temporal derivatives (6 velocity parameters), and the derivatives squared. The motion data for EEG correction was filtered at 11 Hz. While the camera parameters are sampled at 80 Hz, the EEG data is sampled at 5 kHz. Motion and EEG datasets were synchronized to a frequency of 500 Hz; therefore, the camera data was up-sampled (from 80 Hz) and the EEG data down-sampled (from 5 kHz).

Review of EEG data to identify interictal epileptic discharges (IED) and ictal events was made by MS, MC and FM. In cases where no clear IEDs could be identified focal slowing with sharpened transients were marked. The events were marked within Brain Vision Analyzer2 and then exported to be later included in further analysis steps.

### Analysis of Motion Parameters

#### fMRI

Perfect motion correction would result in entirely flat motion realignment parameters and high tSNR. To determine the degree to which the PMC system had removed motion variability in image position we compared the six realignment parameters (exported from SPM) and FIACH noise regressors (representing residual noise components) to PMC. Additionally, we compared FIACH tSNR and number of voxels replaced between subjects and sessions.

To measure motion and its residuals, we calculated the root-mean-square (RMS) for each of the six Euclidian displacements derived from motion measurements (PMC and realignment parameters) and determined the minimum and maximum amplitude on the axis with the largest RMS. Since data for the PMC was acquired at 80 Hz and the realignment parameters (RPs) per every 2.4 s, we down sampled the PMC data to match temporal frequency of the RPs (i.e. per TR).

We additionally assessed the severity of head motion by calculating the total speed for each time point as previously described (Todd et al. 2015):

1 $$s = \sqrt {{{\left( {\frac{{dX}}{{dt}}} \right)}^2} + {{\left( {\frac{{dY}}{{dt}}} \right)}^2} + {{\left( {\frac{{dZ}}{{dt}}} \right)}^2} + {{\left( {\frac{{dRx}}{{dt}}} \right)}^2} + {{\left( {\frac{{dRy}}{{dt}}} \right)}^2} + {{\left( {\frac{{dRz}}{{dt}}} \right)}^2}}$$

The directions of the head translations (in mm) are defined for X (left-right), Y (antero-posterior) and Z (foot-to-head) axes, and Rx, Ry and Rz the corresponding rotations (in degrees). To assure equivalence among rotations and translations, we have assumed a rotational radius of 5.7 cm as previously described (Todd et al. 2015).

We calculated the *averaged speed within a volume* (ASV) as previously defined (Maziero et al. 2020) to summarize the motion recorded by the camera during each fMRI TR into one time point as follows:

2 $$ASV=\frac{{\sum }_{j=1}^{TC}{s}_{j}}{TC}$$

where *TC* is the number of camera sampling points within each fMRI volume and *j* is each sample point acquired by the camera during that volume and *s* is the total speed for each sample acquired by the camera. The ASV is calculated for every fMRI volume and results in a time course of similar length to each fMRI session duration and represents a marker for total motion (Maziero et al. 2020). Finally, we calculated the average of the ASV peaks for each run, therefore we could summarize motion in one measurement for the entire run. The peaks were defined by choosing the motion events that exceeded the mean plus two standard deviations considering the ASV time course of each run. Finally, the maxima of the individual peaks from each fMRI run were averaged. This reflected the average of maximum speeds reached each time patients moved.

#### EEG

We applied different analyses to evaluate the impact of patient’s motion on the quality of the EEG data acquired simultaneously to fMRI.

1. Visual assessment of different approaches of post-processed EEG (BrainVision Analyzer 2, REEGMAS): We visually compared patients’ differently corrected EEGs for each of their EPI sessions, particularly focusing on the parts with heavy motion as well as those where epileptiform activity could be identified.
2. The variance of each EEG run acquired simultaneously to the fMRI data was evaluated. In this study each fMRI slice was acquired in 60 ms and the EEG date were down-sampled to a rate of 500 Hz, which resulted in 30 sampling points for each epoch. We estimated the variance of each sample point across the entire fMRI acquisition for data resulting from the templates used for standard EEG GA correction (EEG_GA_) done with BrainVision Analyzer 2 and REEGMAS (EEG_REEGMAS_). Additionally, we evaluated the variance of each one of the 30 sampling points on the EEG before applying any correction (Raw EEG). The average variance was calculated considering the variances of all electrodes except the ECG channel. The averaged variances were compared among each other using ANOVA with correction for multi-comparisons (p < 0.05).
3. EEG power spectral density (PSD) was calculated by applying the Welch method (pwelch.m function). The spectra were obtained by considering a Hamming window of 3s with 1.5s of overlap. The PSD was normalized at each frequency by sampling rate and the number of samples within each window (dB/Hz). The averaged PSD was calculated considering all the data acquired through a fMRI run. We also calculated two standard deviations above and below the average PSD, we have assumed that points laying outside this range are likely to be related to residual artefacts.

### EEG-fMRI-analysis

#### Pre-processing

The fMRI data was analyzed using Statistical Parametric Mapping (SPM) software version 12. As described previously in work coming from our group (Centeno et al. 2016) after having discarded the first five scans, MRI pre-processing consisted of volume-volume realignment (Friston et al. 1996) as implemented in SPM producing six realignment parameters (RP) followed by retrospective motion correction with FIACH (Tierney et al. 2016b). The FIACH correction produced six noise parameters (values for FIACH tSNR and number of voxels replaced). FIACH detects jumps in signal intensity that are not physiologically plausible whose predominant cause is bulk head movement, this is expressed in the number of voxels that needed to be replaced. The tSNR is the temporal signal to noise ratio, which takes into account the signal changes over time in an fMRI time series (Welvaert and Rosseel 2013). For the fMRI data to conform to the assumptions in gaussian random field theory smoothing should be 3–4 times the voxel size as a minimum (Worsley 2004; Tierney et al. 2016a). In a next step slice timing correction and smoothing with a 12 mm kernel was applied to the FIACH-processed images. Images were analyzed in the subject’s space.

#### EEG fMRI Maps

For creating epileptiform-activity related maps, pre-processed data from fMRI and simultaneous acquired EEG (exported IEDs/sharpened transients) were analyzed in a general linear model (GLM) in SPM. Additionally, we entered the six realignment parameters and six FIACH noise regressors into the GLM as effects of no interest.

Significant epileptiform activity-related BOLD signal changes were tested with an F-test across the three included regressors (canonical hemodynamic response function (HRF) and its two temporal derivatives). Changes in BOLD signal were considered significant above a threshold with p < 0.001 (uncorrected) and a cluster size voxel with a minimum of 5 contiguous voxels (Centeno et al. 2016, 2017).

### Electrical Source Imaging

Only patients with clear-defined spikes captured during their EEG-fMRI sessions had electrical source imaging (ESI) analysis. Spikes were marked manually after removal of scanner artefacts and motion correction. In BrainVision Analyzer 2 marked events were used to calculate the electrical source image using Cartool (Version 3.70, Creative Commons, https://sites.google.com/site/cartoolcommunity/) and followed the procedure described by Vulliemoz et al. (Vulliemoz et al. 2009).

### Comparison of Results to Clinical Localization

In clinical standard practice all patients in presurgical assessment receive a video-EEG-telemetry with surface electrodes, a 3T MRI with epilepsy protocol and neuropsychological testing. The results are then discussed in a multidisciplinary epilepsy surgery meeting and a consensus reached whether the epileptogenic zone had been adequately localized (clinical focus hypothesis) to make an informed decision regarding surgery. In case of discordance of results, non-lesional MRI or suspected bilateral epilepsy further diagnostics can be added, e.g. FDG-PET or ictal-SPECT. If further information about surrounding eloquent areas or more exact localization is needed an intracranial EEG (icEEG) would be acquired, where several electrodes are implanted directly into the brain before performing epilepsy surgery.

To inform us whether EEG-fMRI maps had adequately localized the epileptogenic zone (EZ) we used the clinical focus hypothesis from notes of the latest epilepsy surgery meeting including information from icEEG where it was available. EEG-fMRI results were considered well localizing if localization and lateralization were concordant with the clinical focus hypothesis, or partly concordant if lateralizing to the same hemisphere.

## Results

Ten children with drug-resistant focal epilepsy were recruited and underwent simultaneous EEG-fMRI. Median age of patients was 14 years, median age at onset of epilepsy was 7 years, median duration of epilepsy was 5 years and 40% of patients were female.

All patients received a 10-minute duration EEG recording outside of the scanner and completed 2 of 3 possible EEG-fMRI recording sessions (“runs”). Nine out of ten patients started three EEG-fMRI runs; four runs in three patients were cut short, due to these children not tolerating the scanning process for different reasons.

In nine out of ten patients motion tracking was performed and used for prospective fMRI correction, in one case it was not possible due to technical difficulties. Eight patients had epileptiform activity in their EEG which could be further analyzed; in three of these ten, sufficient well-defined isolated discharges were identified enabling ESI analysis. In the only patient without motion tracking, no epileptiform discharges were identified and therefore they could not be included in either of the analyses. In one further patient with motion tracking, no epileptiform discharges were recorded during the measurements and could be included only in motion evaluations.

### Motion Correction for EEG

We sought to determine EEG quality during prospective motion correction with and without the incorporation of motion information into the retrospective EEG correction procedure.

#### Visual Assessment

**Fig. 1:**
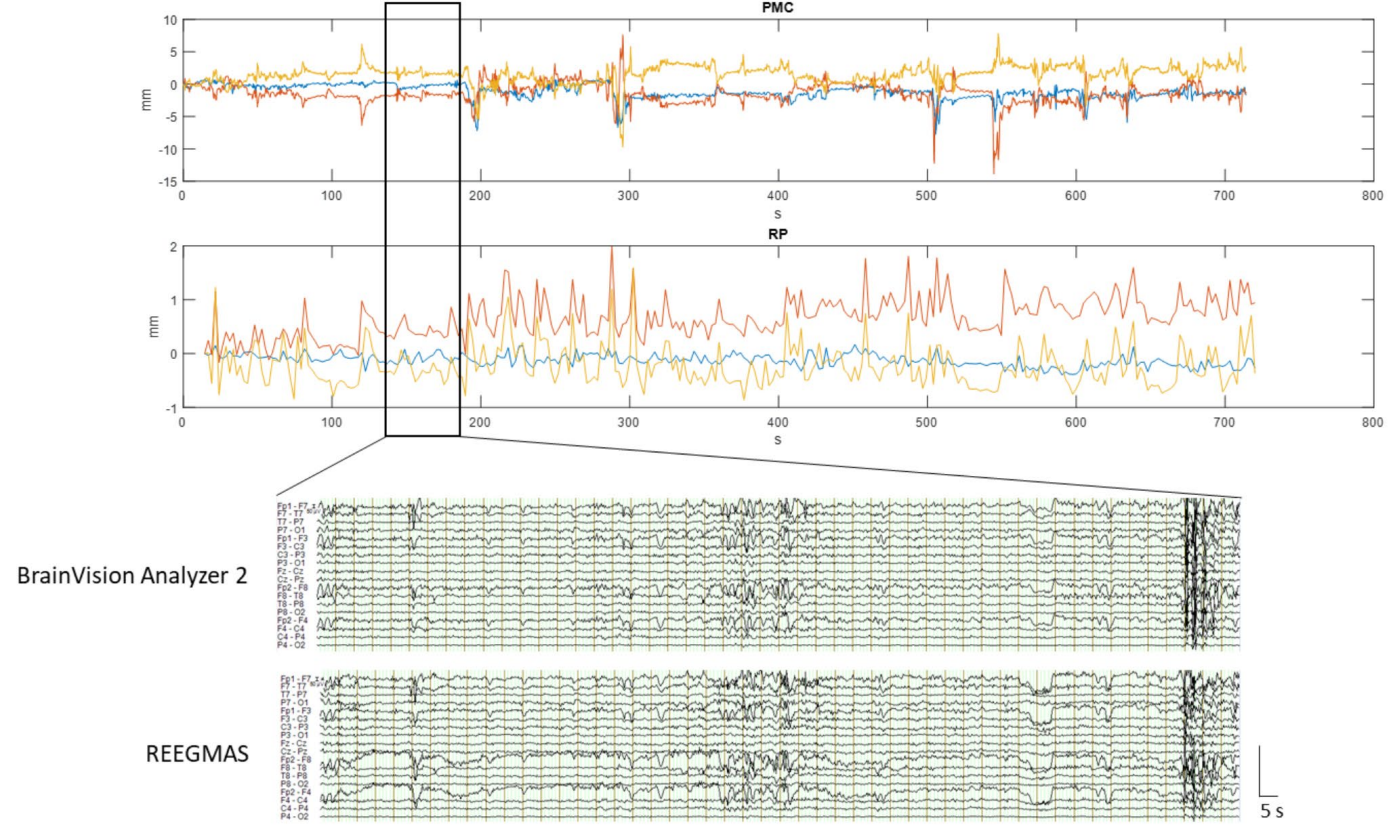
Example of EEG correction from (patient 2, run 2; Brain Vision Analyzer 2 and REEGMAS), 50 s duration, during a period of low motion as indicated by PMC (± 2.5 mm) and RP data (± 0.5 mm)

**Fig. 2:**
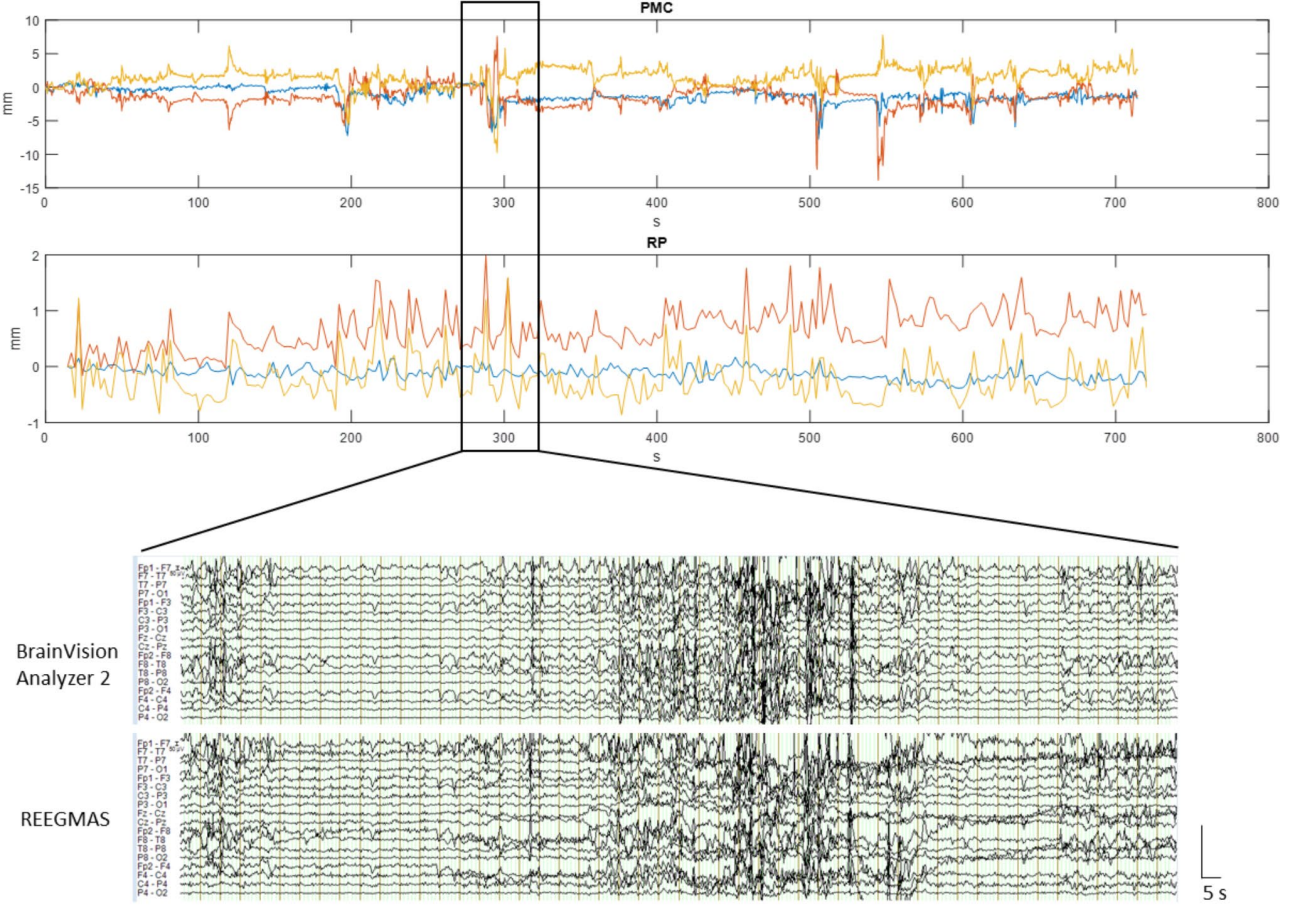
Example of EEG correction (patient 2, run 2; Brain Vision Analyzer 2 and REEGMAS), 50s duration, during a period of high motion as indicated by PMC (± 7 mm) and RP data (± 1.4 mm)

In 8 out of 10 EEGs it was possible to visually identify epileptiform activity during prospective motion correction. In most of those cases interictal epileptiform discharges (IEDs) could be equally well identified by visual assessment in both outside and inside scanner EEG (online resource 2). The results of template artifact corrected EEG and REEGMAS were visually similar during periods of low or high motion (see examples in Figs. 1 and 2). During low motion both retrospective EEG correction methods allowed the visualization and identification of physiological noise and epileptiform discharges if present (Fig. 1).

During periods of high motion, both methods showed significant limitations with periods of EEG data corruption that precluded event detection (Fig. 2). An estimate of the ability of the camera system to track and therefore prospectively correct the subject motion can be obtained by comparing the motion measured by the camera and the (uncorrected residual) motion detected by realignment of fMRI images.

Comparing these, there is a five-fold reduction in motion from the prospective motion during low and high motion in the given examples (Figs. 1 and 2). In Fig. 2 it seems that due to the large extent of motion (± 7 mm in PMC data) there was significant residual motion visible in the realignment motion data. Translations are likely to result in GA artefact changes, while residual uncorrected rotations of this magnitude could not recover the EEG data.

**Fig. 3:**
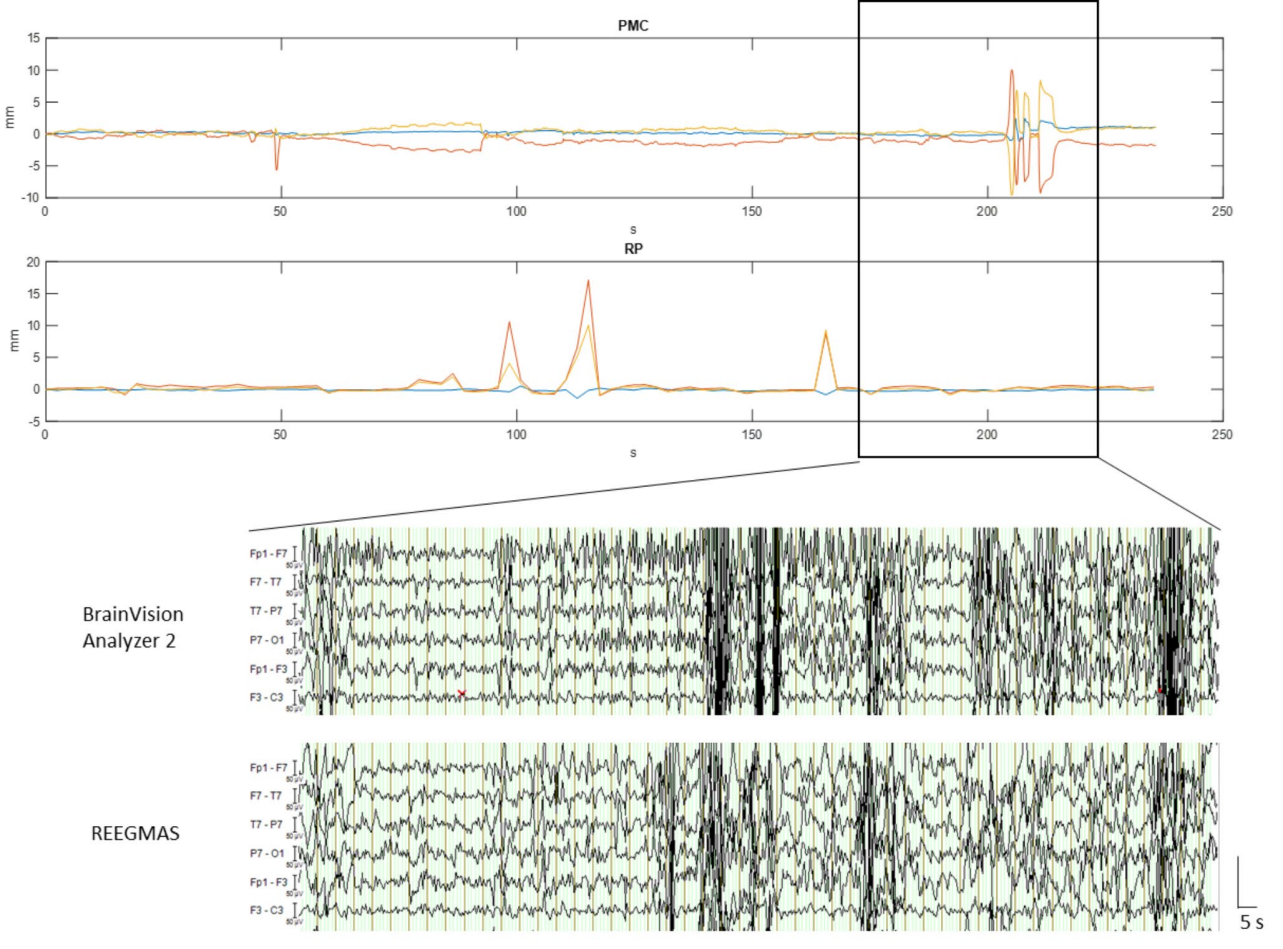
Motion traces of fMRI run 2 of patient 4; upper panel shows head motion in the scanner over time as detected by the PMC camera; lower panel shows SPM realignment parameters over time corresponding to the PMC data. Example of EEG correction (Brain Vision Analyzer 2 and REEGMAS), 50s duration, during a period of high motion as indicated by PMC (± 10 mm) and RP data (± 1 mm)

In a further example with high movement detected by the PMC camera, but corresponding flat RP parameters (Fig. 3), REEGMAS has recovered the data slightly better than standard AAS correction, though significant motion-related artefacts remain.

#### Variance of EEG data

**Fig. 4:**
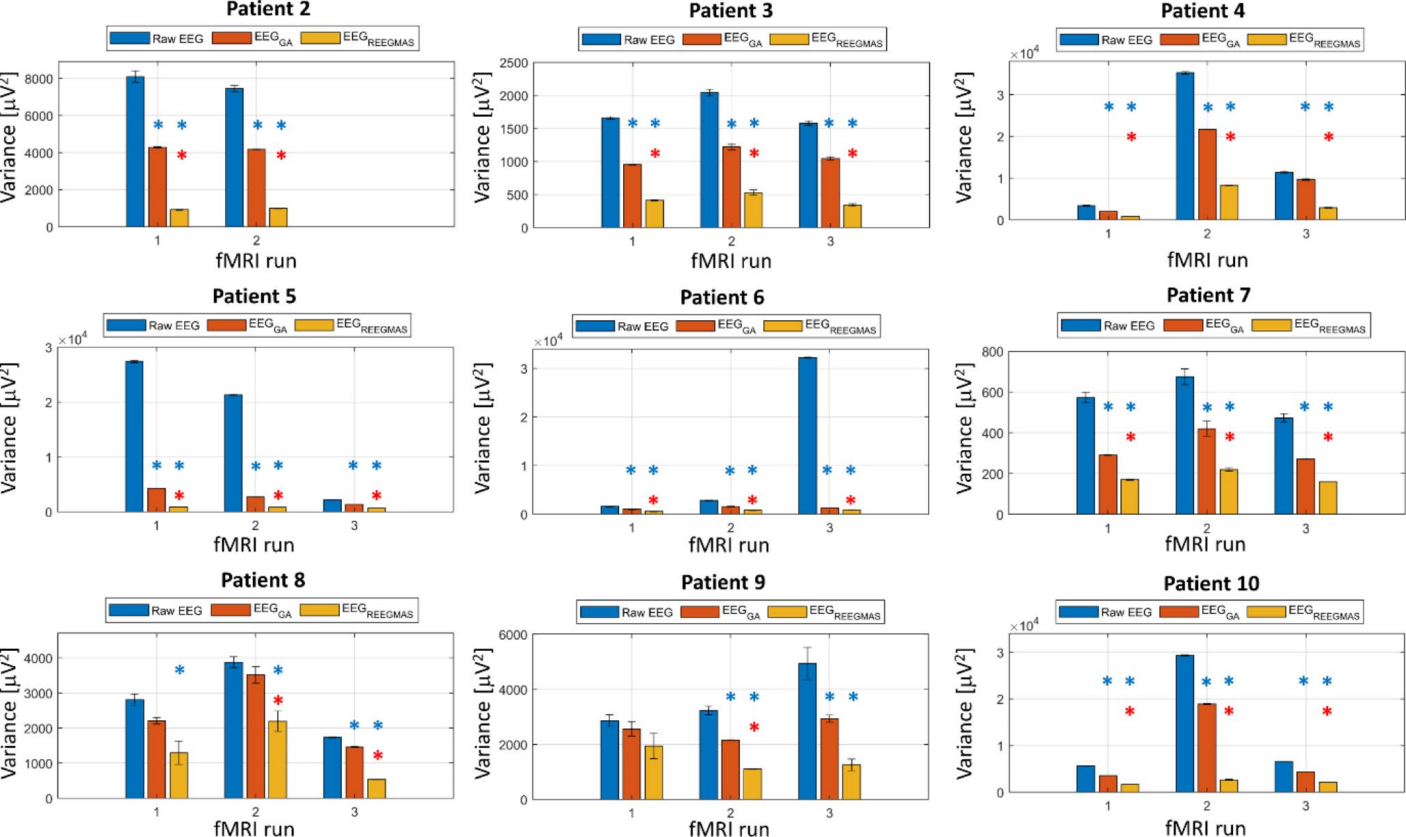
Averaged variances of raw EEG data and EEG data post correction using the different correction methods for data acquired simultaneously to prospective motion corrected fMRI. For each patient and fMRI run the variance was calculated for each slice TR and from these the average variance was calculated for the raw EEG (blue), EEG corrected for GA using standard AAS (red) and REEGMAS (yellow) considering all electrodes but excluding ECG. The standard error on the mean variance is presented by the error bars. The blue * highlights templates with averaged variance significantly smaller than the variance of the Raw EEG and the red * highlight runs where the average variance for the data corrected by REEGMAS are significantly smaller than the variance of the data resulting from standard AAS GA correction

The variance on the data following the correction with REEGMAS was significantly smaller (p < 0.05, corrected for multi-comparison) than the variance on the raw EEG for 25/26 runs (all but patient 9, run 1) (Fig. 4).

The variance on the data following standard AAS template for GA correction was significantly smaller (p < 0.05, corrected for multi-comparison) than the variance on the raw EEG for 23/26 runs (all runs of all patients with the exception of patient 8, runs 1 and 2 and patient 9, run 1) (Fig. 4). The variance on the data following correction by REEGMAS was significantly smaller (p < 0.05, corrected for multi-comparison) than the variance present on the data corrected by standard AAS in 24/26 runs (for all runs of all patients but patient 8, run 1 and patient 9, run 3) (Fig. 4).

#### EEG Power Spectral Density (PSD)

**Fig. 5:**
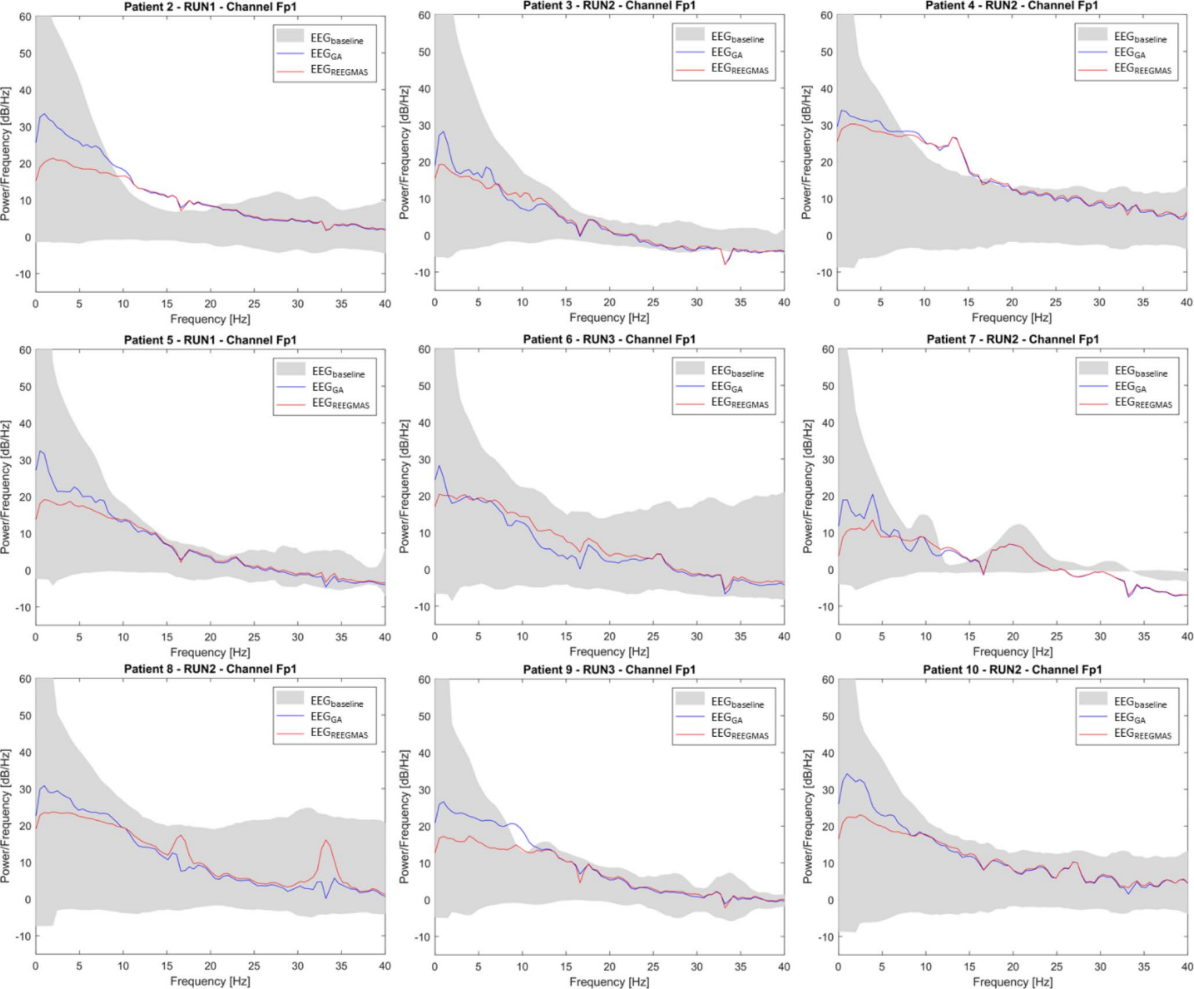
The mean power spectral density (PSD) at electrode Fp1 for each patient. The mean PSD for each patient is presented for the EEG run with the highest averaged variance for the Raw EEG data (Fig. 5). The shaded grey area represents two standard deviations from the mean baseline spectra obtained from EEG data from outside the MRI scanner. The blue curve refers to the mean power spectra of the EEG acquired in-scanner but not corrected by REEGMAS and the red dashed line to the same data corrected by REEGMAS. Electrode Fp1 is shown due to its clinical relevance for this patient population

The EEG data correction using REEGMAS exhibited lower spectral power at low frequencies (< 10 Hz) in comparison to the data corrected by standard AAS in 24/26 runs (all patients except patient 4, run 2 and patient 6, run 3) (Fig. 5). The spectral power between 10 and 25 Hz frequencies was increased by REEGMAS correction compared to template correction for patient 3 (all runs), 5 (run 3), 6 (run 3) and 8 (run 2). An increase on power on the frequencies 16.3 and 33.2 Hz (harmonic) was observed for patient 8, run 2 (Fig. 5).

The power for EEG data corrected for standard AAS and REEGMAS was above or below the two-standard deviation of the baseline EEG for patients 2 (run 1 and 2), 4 (run 2), 7 (all runs) for a range of frequencies between 10 and 20 Hz (patients 2, 4 and 7) and for frequencies above 25 Hz (patient 7).

### Motion-statistics for fMRI Metrics

**Fig. 6:**
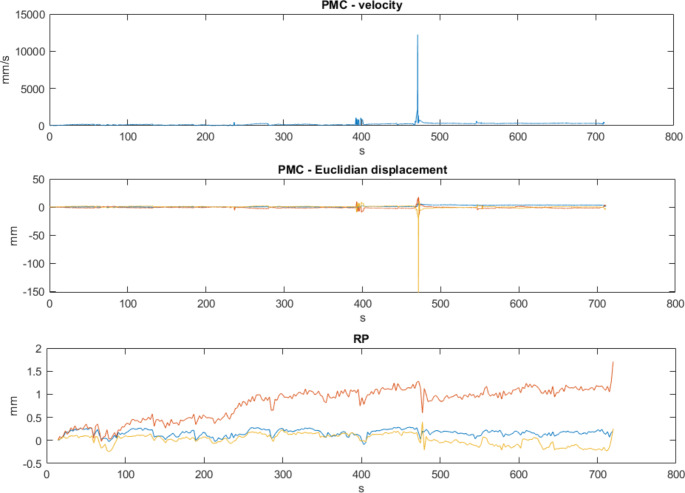
PMC velocity in mm per second and Euclidian displacement as detected by the PMC-camera system and realignment parameters (RP) of the first fMRI run of patient 5. At approx. 480 s a very large peak can be seen. Occurring mostly in the z-axis

Movement during EEG-fMRI sessions did not only differ between subjects but also within subjects across sessions. Three of ten patients moved less than the average of the group over all their sessions (mean RMS velocity < 1.5 mm/s), which in this context could be regarded as low movement. The other seven patients moved in varying degrees over their sessions, which is also expressed by their average ASV peak (Table 1). In some patients (e.g. patient 5, Fig. 6) there seem to have been a number of sudden, short fast movements during the scan (in the example shown in Fig. 6 mostly in the z-axis). The mean velocity of these events would indicate a high motion rate (Fig. 4).

**Table 1:** Overview of patient’s RMS of PMC Euclidian displacement and velocity and ASV Peaks

Patient	Age	Session	Euclidian Displacement [mm]				Velocity [mm/s]			ASV Peaks [mm/s]	
			Median	Mean	SD	Max	Mean	SD	Max	Mean	N peaks
2	11	EPI1	4.31	7.05	8.51	1.30	2.94	3.55	103.70	3.02	12
		EPI2	4.53	7.34	8.94	1.43	3.06	3.72	114.53	3.52	11
3	14	EPI1	2.15	3.09	2.93	0.43	1.29	1.22	34.04	2.03	5
		EPI2	2.19	3.16	2.98	0.28	1.32	1.24	22.17	0.5	2
		EPI3	2.15	3.14	2.99	0.23	1.31	1.24	18.06	2.93	4
4	14	EPI1	2.67	3.71	3.75	0.47	1.55	1.56	37.68	1.01	8
		EPI2	2.15	3.14	2.99	1.22	1.76	3.77	97.40	7.41	1
		EPI3*	3.45	6.06	10.02	1.80	2.53	4.17	143.65	3.7	3
5	14	EPI1*	2.58	5.80	212.98	132.62	2.42	88.74	10609.35	9.15	5
		EPI2*	2.72	5.88	214.94	135.11	2.45	89.56	10808.98	8.25	7
		EPI3	2.67	3.32	2.68	0.31	1.39	1.18	24.72	1.64	13
6	15	EPI1	2.22	3.07	2.92	0.31	1.28	1.22	24.73	0.96	5
		EPI2	2.31	3.19	3.26	0.42	1.33	1.36	33.97	1.52	5
		EPI3	2.61	3.45	2.84	0.18	1.44	1.18	14.28	0.76	4
7	17	EPI1	1.40	1.63	1.10	0.11	0.68	0.46	8.85	0.67	2
		EPI2	1.61	2.02	1.56	0.13	0.84	0.65	10.43	1.24	7
		EPI3	1.38	1.65	1.13	0.09	0.69	0.47	7.41	0.63	6
8	15	EPI1	2.64	3.34	2.95	0.34	1.39	1.23	27.15	2.09	11
		EPI2	3.13	4.02	3.46	0.31	1.68	1.44	25.20	2.8	12
		EPI3	2.41	2.99	2.53	0.39	1.25	1.05	31.55	2.15	11
9	14	EPI1*	3.22	4.49	4.74	1.35	1.87	1.98	108.19	2.76	12
		EPI2	3.55	4.97	4.61	0.33	2.07	1.92	26.63	3.16	10
		EPI3	4.04	5.59	5.17	0.55	2.33	2.16	43.75	3.76	11
10	11	EPI1	2.50	3.30	2.89	0.33	1.38	1.20	26.13	0.74	10
		EPI2*	2.51	5.37	182.83	161.93	2.24	76.18	12954.04	6.45	6
		EPI3	2.80	3.84	3.35	0.29	1.60	1.40	23.26	0.62	9

SD: standard deviation; Max: maximum; ASV Peaks: averaged speed and number of peaks within a volume. *sessions with very high velocities marked here are most likely due to the PMC camera losing the tracker rather than actual movement happening

**Fig. 7:**
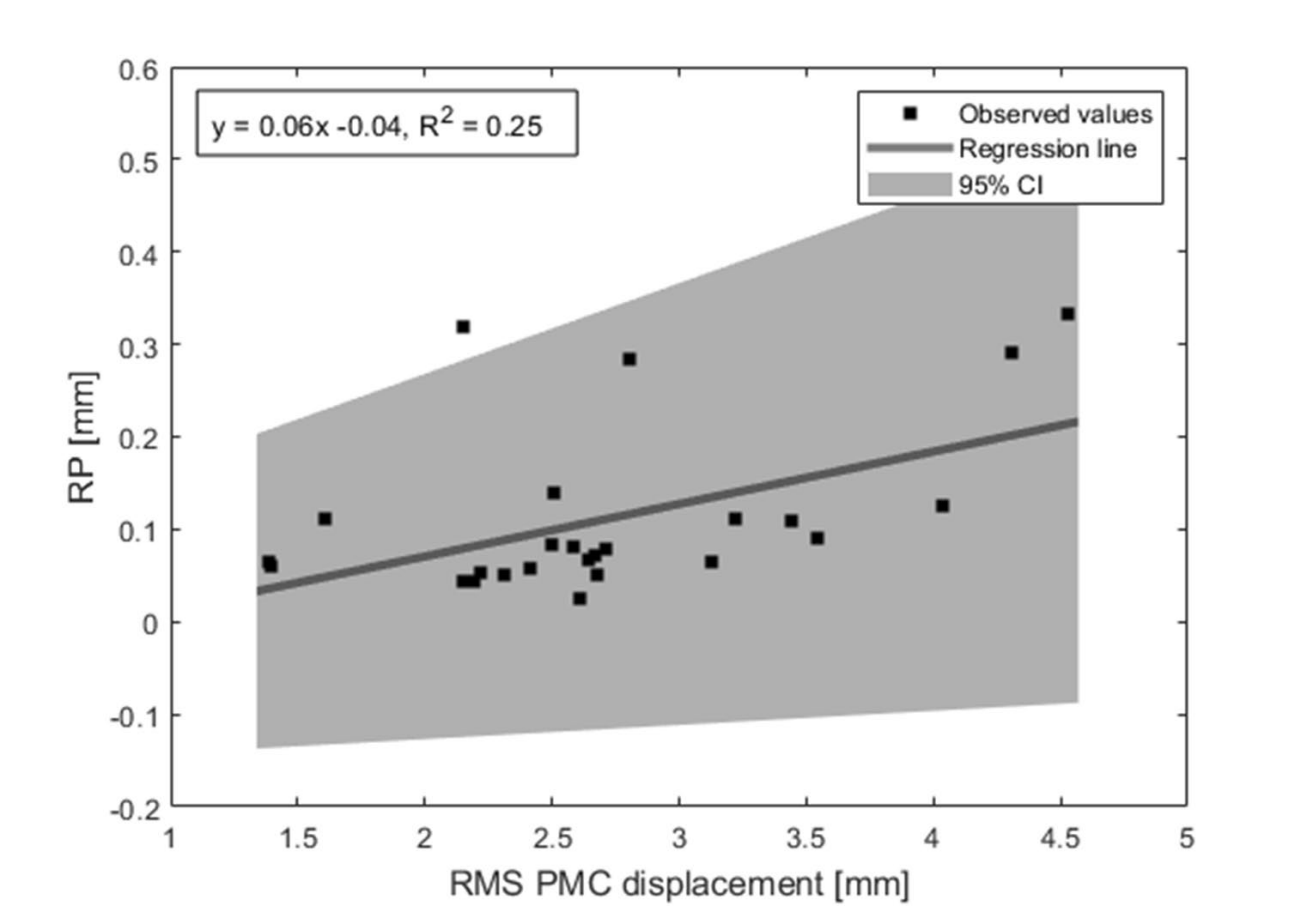
Linear Regression of median root mean square (RMS) of realignment parameters (RP) and RMS of Euclidian displacement of measured motion from the prospective motion correction (PMC); 95% CI: 95% confidence interval

The effect of fMRI prospective motion correction should be to minimize the spatial displacement of images through time. To determine the effectiveness of the prospective correction we compared the measured motion from the PMC system to both residual fMRI motion realignment and residual temporal signal discontinuities larger than typical BOLD signal changes. To analyze the impact of the different motion correction steps further we looked at the relationship between PMC, RP, FIACH tSNR and number of voxels replaced by FIACH. Since we wanted to assess the amount of movement without being influenced by extreme values, we decided to look at median values for this analysis. Linear regression of the root mean squares (RMS) of the median Euclidian displacement of the PMC data and of the RP from SPM (Fig. 7) showed an F-ratio of 8.0 (F significance = 0.009), meaning that the variability of the data can be well explained by the model. The gradient of the model is 0.06, R^2^ = 2.5 and adjusted R^2^ = 2.2 (p = 0.009). This indicates that residual motion effects from the prospectively corrected data are related to subject head motion. The difference in magnitude of motion lies between 1.5 and 4.5 mm for the PMC and 0.04 to 0.35 mm for the RP. This means that after prospective motion correction less than 10% residual motion remains. Though when motion was high (> 4.3 mm) residual motion was high as well.

Figure 3 shows the temporal course of motion parameters of fMRI run 2 of patient 4, that corresponds to the outlier outside the confidence interval (2.15, 0.32) in Fig. 7. There are three large peaks of residual motion in the RP data, in the corresponding camera data of actual motion no peaks are visible. Prior to each peak there is continuous motion in the PMC data ending just before the peak in the RP data, which could indicate overcorrection or delay in the end of the correction. In the same session there is large motion towards the end in the PMC data and flat corresponding RP parameters, indicating effective motion correction by PMC system during this period.

**Fig. 8:**
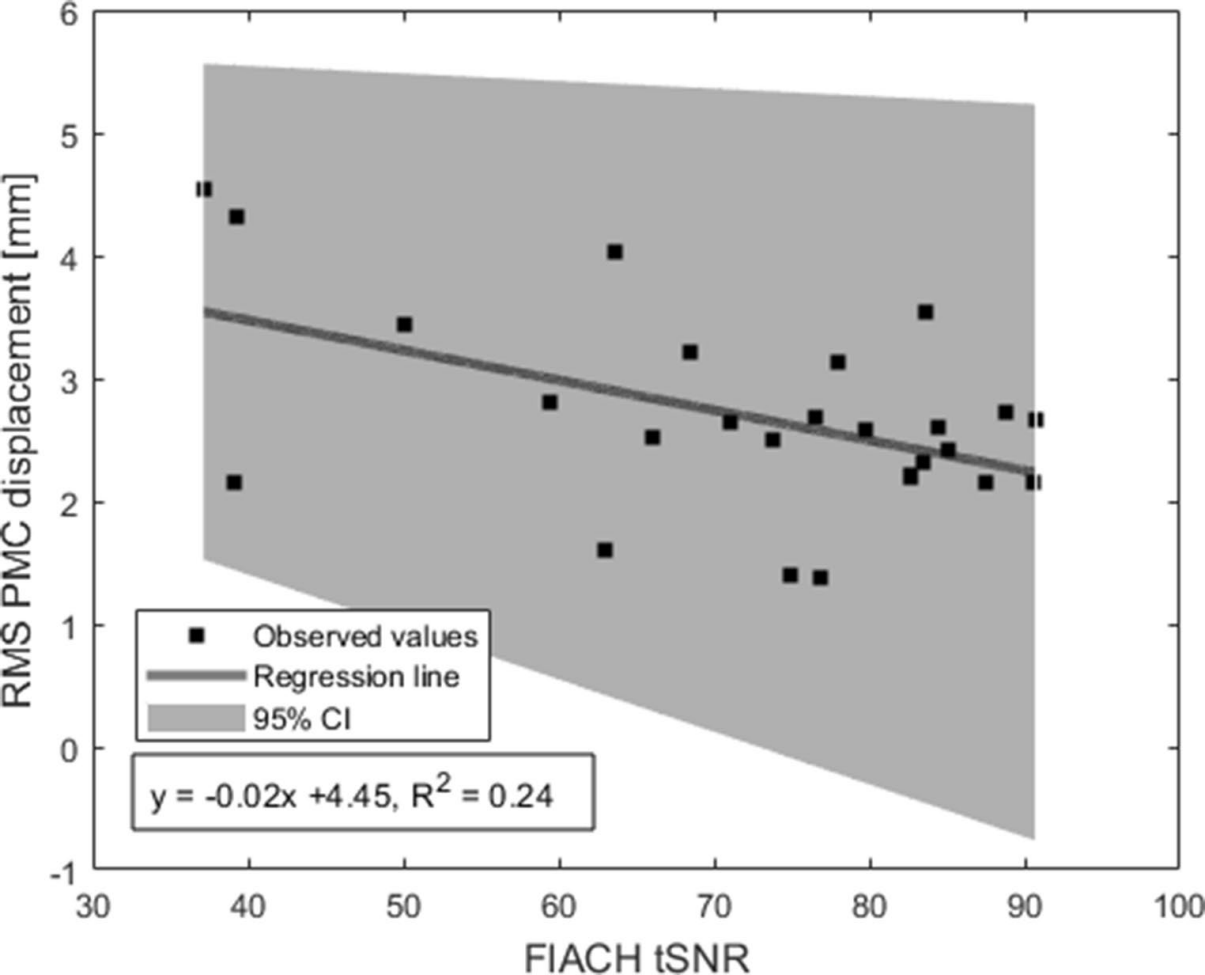
Linear Regression of FIACH tSNR and median RMS of Euclidian displacement of prospective motion correction (PMC); 95% CI: 95% confidence interval

The relationship between FIACH tSNR and PMC parameters was also significant (F = 7.6; R^2^ = 0.24, adjusted R^2^ = 0.21; p = 0.01), showing increased tSNR values for low motion and reduced tSNR when motion is greater (Fig. 8), indicating good prospective correction.

**Fig. 9:**
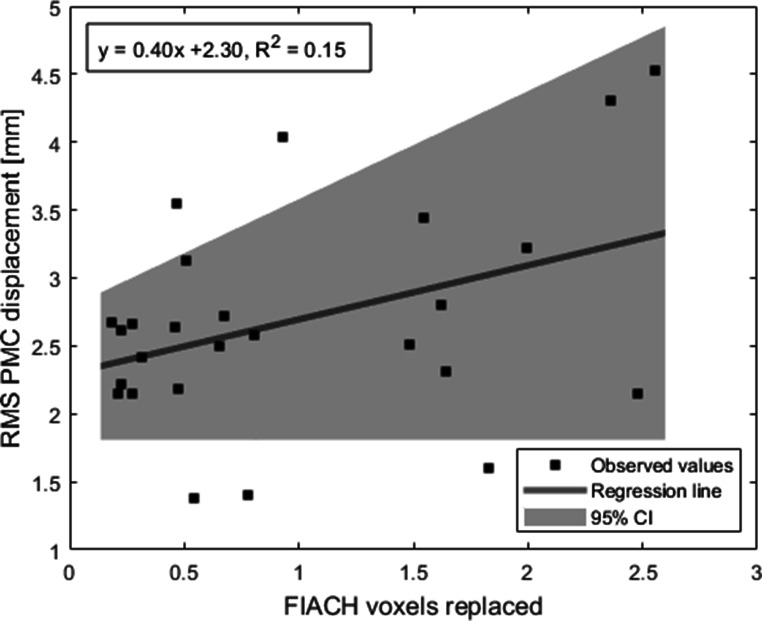
Median RMS of Euclidian displacement of prospective motion correction (PMC) over FIACH voxels replaced; y = slope x intercept; 95% CI: 95% confidence interval

There was a weak linear relationship between PMC parameters and number of FIACH replaced voxels (F = 4.3; R^2^ = 0.15, adjusted R^2^ = 0.12; p = 0.05) (Fig. 9).

### Localization of the Epileptogenic Zone Through EEG-fMRI

**Fig. 10:**
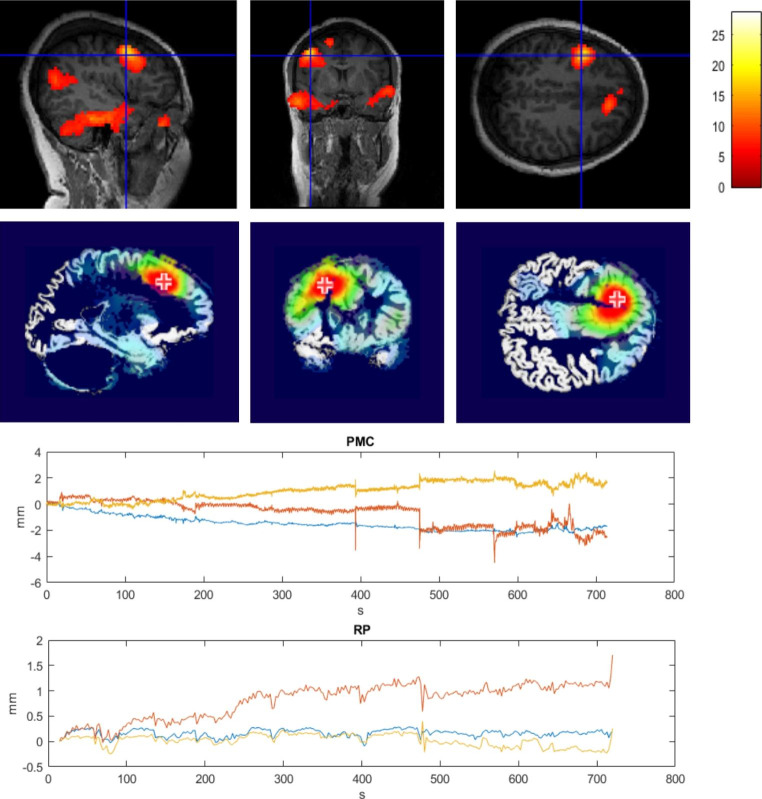
fMRI (upper panel) and ESI results (second panel) of patient 4 showing a concordant left frontal focus; the cross-hair/ cross marks the point of maximal activation/ electrical activity. The lower two panels show the amount of movement in the concordant fMRI run as recorded by the camera before (PMC) and after (RP) prospective motion correction

Seven patients showed significant epileptic activity-related BOLD activation on EEG-fMRI maps, in five of them EEG-fMRI results were concordant with the clinical hypothesis. In all three patients in whom ESI was feasible, ESI localization was concordant or partly concordant with EEG-fMRI results. An overview of EEG-fMRI and ESI results in comparison to the clinical hypothesis made after telemetry, structural MRI and FDG-PET can be seen in Table 2 and a visual example of fMRI and ESI results in Fig. 10 (further ESI results are presented in Online Resource 1).

**Table 2:** Results of EEG-fMRI and ESI compared to each other and clinical hypothesis; patients ranked by amount of movement

Patient	PMC Median Euclidian displacement [mm]	EEG-fMRI concordance with clinical hypothesis	EEG-fMRI concordance with ESI	Clinical hypothesis after telemetry	EEG-fMRI findings	ESI
2	4.42	discordant for exact focus; concordant for side	-	right anterior quadrant	right posterior temporal	-
9	3.60	-	-	right frontal	no conclusive results	-
8	2.73	concordant	partly concordant	left frontal/anterior quadrant	bilateral with left frontal maximum	bilateral, with left fronto-temporal maximum
4	2.76	concordant	concordant	left mesial frontal	left frontal	left frontal
5	2.66	concordant	-	left anterior quadrant	left frontal	-
10	2.60	concordant	concordant	bilateral frontal with left maximum	bilateral with left frontal maximum	bilateral with left frontal maximum
6	2.38	discordant	-	left posterior temporal/parietal	right fronto-temporal	-
3	2.16	concordant	-	central/ left frontal	central over all sessions; left anterior temporal one session	-
7	1.46	-	-	left hemisphere	None (no IEDs)	-

PMC: prospective motion correction; EEG: electroencephalography; fMRI: functional magnetic resonance imaging, ESI: electrical source imaging. Patient 1 is not featured in this table, since no prospective motion correction was available, and no epileptiform activity was recorded during EEG-fMRI

Finally, five (patients 3, 4, 5, 6 and 9) out of 10 patients were offered invasive EEG-recordings to localize the seizure onset zone using SEEG. Four patients (patients 3, 4, 5 and 6) went through with the procedure. In one patient (patient 5) this resulted in epilepsy surgery using laser interstitial thermal therapy (LiTT). The patient showed a significant improvement of the seizures (ILAE 3)(Durnford et al. 2011) one year after surgery. In the other three patients no clear surgical target could be identified through SEEG. The remaining five patients were not considered suitable candidates for SEEG or surgery.

## Discussion

Simultaneous EEG-fMRI can add valuable information on the localization of the epileptogenic focus in patients with drug-resistant focal epilepsy undergoing presurgical assessment (Markoula et al. 2018). This has been shown repeatedly in epilepsy patients using both scalp (Salek-Haddadi et al. 2006; Chaudhary et al. 2012; Centeno et al. 2017; Kowalczyk et al. 2020; Koupparis et al. 2021) and intracranial EEG (Chaudhary et al. 2016) simultaneously recorded to fMRI.

Head motion artefacts remain the most important confounder when trying to reliably detect IED related BOLD responses. Retrospective metrics to remove motion artefacts from fMRI often remove a large percentage - in some cases all - of the relevant data (Power et al. 2014). Therefore, removing motion effects from fMRI and EEG data while still retaining the relevant clinical information remains an ongoing challenge (Zaitsev et al. 2017; Power et al. 2014; Maziero et al. 2016).

Motion artefacts in MRI are particularly challenging in pediatric populations (Poldrack et al. 2002; Brown et al. 2010), though it has been shown that EEG-fMRI can be applied successfully in that group (Centeno et al. 2016; Moeller et al. 2013).

In this study we demonstrate the effect of combined prospective and retrospective motion correction on the results of simultaneous EEG-fMRI obtained from 10 unsedated children with drug-resistant focal epilepsy. Even though the general amount of head movement in the scanner was high, relevant clinical information was still recovered from the motion-corrected EEG-fMRI.

### Quality of EEG Correction

Despite the traditional assumption that prospective motion correction of fMRI precludes EEG artifact correction, we have shown that EEG can be recovered and motion information incorporated into the correction. The impact of this is contingent on the quality of the tracking information and level of motion.

Visually we did not detect relevant differences in the quality of corrected EEG comparing AAS vs. REEGMAS, though previously reported results for REEGMAS had been better (Maziero et al. 2016, 2021). We used the same parameters for both EEG correction approaches. However, it is possible that some minor differences between implementations remain.

It should be noted that the baseline EEGs for comparing Power Spectral Density obtained out-of-scanner have strong signals related to motor activity and muscle-related artefacts which contrasts with previously published data (Maziero et al. 2016), where very clean EEG data with a well-defined alpha rhythm and without any strong physiological-related artefact in healthy adults were obtained and analyzed. This potentially biases assessment based on Root Mean Square Error (RMSE) as has been used and published previously (Maziero et al. 2016). When computing RMSE on the current data we noticed that there were many runs where the RMSE was reduced at low frequencies for REEGMAS compared to AAS, making AAS appear more similar to baseline levels (Fig. 5). This may give the false impression that AAS is providing better correction than REEGMAS whereas this is at least in-part related to noise sources in the physiological baseline recordings. Instead of an RMSE analysis we looked at the variance of the gradient artefacts across all epochs of each slice, because it is not affected by baseline data and therefore a better metric to compare AAS vs. REEGMAS EEG corrections. This demonstrated that the EEG variance following correction by REEGMAS was significantly smaller than when corrected by standard AAS. We note that REEGMAS has a greater reduction in EEG spectral power in the ~ 0-10 Hz range owing to the choice of a filter on the motion data (11 Hz). Although a greater range of frequencies could be used in general head motion is predominantly within this range.

The activity of interest (epileptiform activity) could equally been identified using both methods of EEG correction, meaning that the REEGMAS method does preserve this EEG feature for visual identification and it is reasonable to use in patients with epilepsy.

### Quality of fMRI Correction

Prospective motion correction of fMRI can be utilized for EEG-fMRI. We demonstrated a substantial improvement in fMRI data quality after prospective motion correction in the context of subject motion.

Measures like realignment parameters of fMRI images, as well as FIACH tSNR and replaced voxels can be used to give an estimate of the ability and quality of prospective motion correction. In theory, perfect correction by PMC would lead to flat RP, high FIACH tSNR and zero replaced Voxels. However, the PMC camera cannot correct for the motion-related changes in B0 field homogeneity – for that dynamic shimming would be needed – meaning that even under perfect conditions some voxels would still be replaced because of susceptibility artefacts (Boegle et al. 2010).

We have shown that residual motion effects from the prospectively corrected data are related to subject head motion: After PMC less than 10% residual motion remained, the degree of residual motion in the images was related to the amount of motion measured from tracking. In general, this suggests that RPs should be included into the fMRI model as confounds.

The very high velocities detected in only one axis as for example displayed in Fig. 6 could be the result of the PMC camera losing the tracking signal, which led to a short peak in motion metrics, rather than actual movement. Alternatively, it is possible that the patients played with the bite bar in their mouths and created these events.

There are a number of retrospective motion correction approaches for fMRI where a set number of regressors (Power et al. 2014) or one regressors for each motion event (Lemieux et al. 2007) is added to the general linear model and through exclusion of motion-contaminated volumes the effect of motion in the overall dataset reduced. The strength of these approaches lies with the robustness of the results, though data cannot be recovered and particularly in subjects that move more the degrees of freedom in those datasets can be greatly reduced (Zaitsev et al. 2015).

Currently two types of PMC can be distinguished: Head motion is tracked by either a MR navigator (working in image space or *k*-space) or alternatively through a MR compatible external tracking device (stereo camera systems, miniature RF probes, in-bore camera systems or ultrasound systems) potentially with an added device mounted on the subject (Zaitsev et al. 2015) as we have used in this study. The general advantages of using PMC are that it reduces spin-history effects and simplifies the approach by not requiring sequence-specific modifications needed for MR navigators

. Once setup it is flexible in its application since it is valid for all current field strengths on most clinical scanners (Maclaren et al. 2013) and for fMRI in particular it has been shown to reduce motion induced false positive activations (Schulz et al. 2014).

Limiting factors of PMC are among other things the accuracy and precision of the tracking system, besides gradient imperfections and B0 inhomogeneities, this is strongly influenced by a secure attachment of the marker, which in turn is contingent on a reasonable comfortability for the subject/patient (Maclaren et al. 2013). The marker was one of the major practical difficulties for this study. Firstly, children had to make two visits for the individual dental retainer to be fitted before the actual scanning session. Second, several children were observed to start to play with the dental retainer in their mouths leading to the decoupling of detected and actual motion.

Previous studies investigating prospective motion correction during fMRI or simultaneous EEG-fMRI did so in young healthy adults with motion and no-motion conditions (Todd et al. 2015; Maziero et al. 2016, 2020), which is good for a proof of concept but far removed from the clinical reality. Here, it was established for the first time that significant clinically relevant data recovery is possible utilizing prospective motion correction techniques for EEG-fMRI data. Therefore, as motion tracking technology advances and reduces the practical limitations related to marker fixation prospective motion correction can play a significant role.

### Validity of Localization of Epileptogenic Focus Through EEG-fMRI After Prospective Motion Correction

To be able to reliable obtain good image quality in children they often need to be sedated throughout the procedure (Siniatchkin et al. 2018). It has been shown to work in unsedated children by enhancing tolerability for children e.g. through showing them a cartoon, which led to reduced in-scanner movement (Centeno et al. 2016). Though the latter might not always work, particularly in smaller children or those with additional mental disability. Sedation during EEG-fMRI is not practical if the aim is to use it to localize the epileptogenic zone (EZ) due to reduced epileptiform activity under sedation. Being able to use prospective motion in restive patients could greatly improve diagnostic yield and quality.

Most studies so far used retrospective motion correction to be able to receive high image quality in MRI in unsedated children, both for structural (Vecchiato et al. 2021; Kecskemeti et al. 2018) and functional MRI (Centeno et al. 2017).

In previous studies, the test yield (percentage of patients with significant BOLD activations) was reported as 29–89% and the localizing value of EEG-fMRI as 44–74% (Centeno et al. 2017; Salek-Haddadi et al. 2006; Grouiller et al. 2011). In our study seven out of 10 patients had a significant cluster on EEG-fMRI maps and those results were concordant with the clinical hypothesis regarding region and hemisphere in five cases (71%) and in one other case concordant with the hemisphere only (14%). Five patients received a recommendation for SEEG, four of which did undergo the procedure. Finally, one child underwent epilepsy surgery, with a good postoperative seizure outcome (ILAE 2) 2 years after surgery. In these previous studies a number of patients are excluded based on data corrupted by movement artifact. Overall, PMC was effective because despite large in-scanner movement in some of the children, we achieved concordant EEG-fMRI localization results in 6/7 of patients. To be able to make a definitive statement about concordance of EEG-fMRI results, relevant overlap of the EEG-fMRI results with the resected area and seizure freedom after surgery is the gold standard. This issue has been discussed in the past as surgical outcome is often not available (Salek-Haddadi et al. 2003). Nonetheless, it is a limitation of our study that only one patient underwent surgery and we had to rely on the clinical focus hypothesis as reference point in all other patients to judge concordance. Three out of four patients that underwent SEEG did not get a recommendation for surgery. EEG-fMRI results were concordant with the clinical focus hypothesis and had a spatial overlap with SEEG coverage in 2/3, but SEEG was inconclusive. Inconclusive SEEG results may put into question the validity of the prior clinical focus hypothesis, but one cannot falsify this hypothesis with certainty due to limitations in spatial coverage of SEEG. It is possible that within a large irritative zone (IZ) the seizure onset zone (SOZ) was missed, which can happen if SEEG electrodes are only a few millimetres off target (Zijlmans et al. 2019). Having these limitations in mind ESI was performed as an additional independent test where possible, to verify our EEG-fMRI results. Here we could show concordance of ESI with EEG-fMRI in 2/3 and partly concordance in 1/3. Concordance between EEG-fMRI and ESI means that we have two independent tests giving a localisation of the same interictal events. This meets our objectives of showing that the test has worked in that it has localised those events captured during the recording even during high motion levels. However, there remain potential limitations related to the possible discrepancies between the IZ identified at the time of recording and the SOZ defined by SEEG.

## Conclusion

We present for the first-time feasibility of combined prospective and retrospective correction used on EEG-fMRI data in a pediatric cohort with drug-resistant focal epilepsy as a tool to localize the epileptic focus. We succeeded in doing so despite the traditional assumption that PMC precludes EEG artifact correction. Furthermore, a comparison of two retrospective EEG approaches (AAS and REEGMAS) led to equally good results. Our data shows, that the combination of both prospective and retrospective motion correction enables recovery of otherwise lost data and leads to clinically relevant results through its added information on the localization of the epileptogenic zone.

## Electronic Supplementary Material

Below is the link to the electronic supplementary material.

Supplementary Material 1

Supplementary Material 2

## Data Availability

The data that support the findings of this study are available from the corresponding author upon reasonable request due to privacy issues of clinical data. A formal data sharing agreement is needed.
