# Supplementary material for: Camera-based Prospective Motion Correction in Paediatric Epilepsy Patients Enables EEG-fMRI Localization Even in High-motion States
Source: Brain Topogr. 2023 Mar 20;36(3):319–37. doi: 10.1007/s10548-023-00945-0 (PMC10164016; doi:10.1007/s10548-023-00945-0)
Supplement: Supplementary file 1 — Supplementary Material 1 [file 10548_2023_945_MOESM1_ESM.docx]

**Supplementary figure 1** – *Combined fMRI and ESI results*


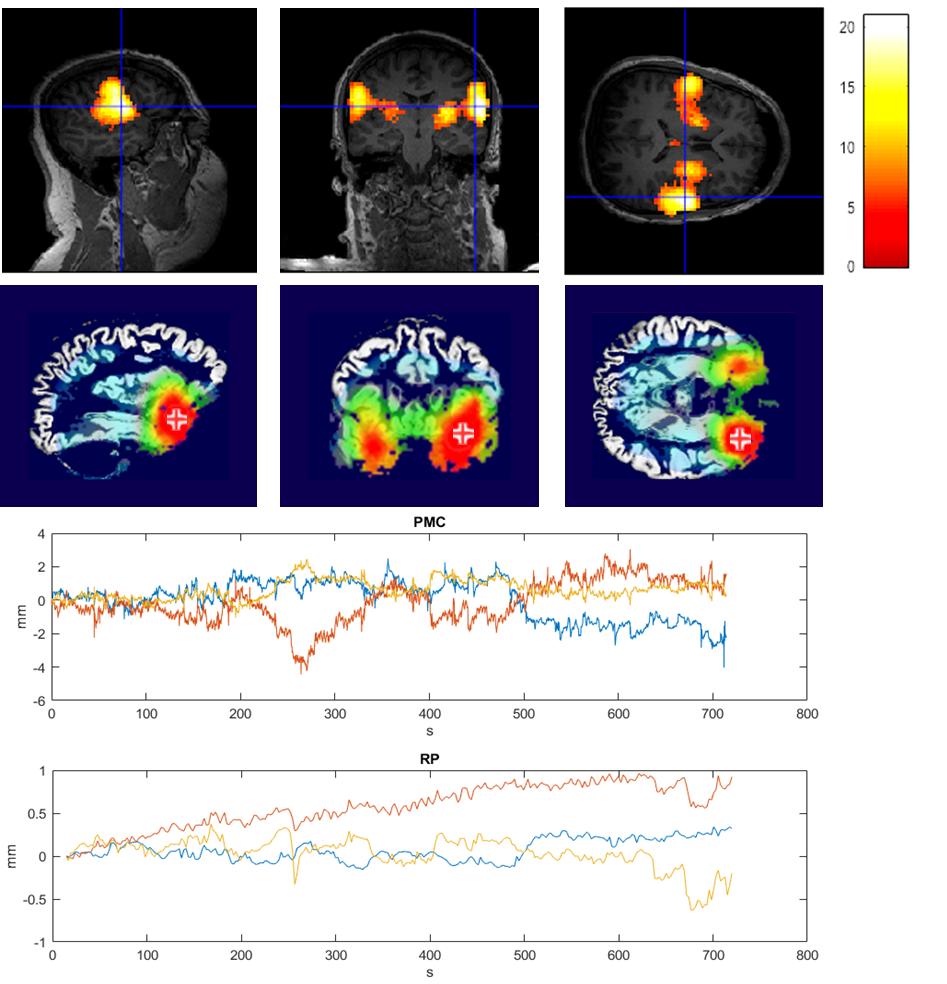


fMRI (upper panel) and ESI results (second panel) of patient 8 showing a bilateral focus with left frontal maximum on fMRI and partly concordant bilateral fronto-temporal focus with maximum on the left on ESI; the cross-hair/ cross marks the point of maximal activation/ electrical activity. The lower two panels show the amount of movement in the concordant fMRI run as recorded by the camera before (PMC) and after (RP) prospective motion correction.


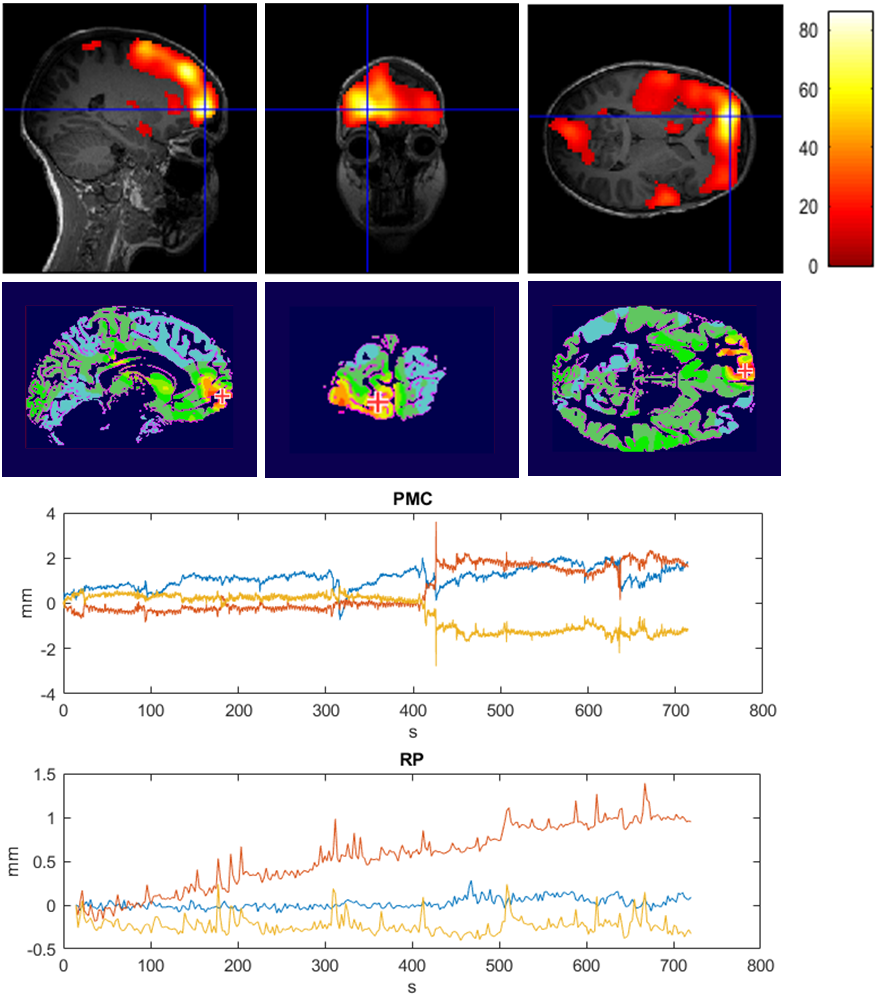


fMRI (upper panel) and ESI results (second panel) of patient 10 showing a bilateral focus with left frontal maximum on fMRI and concordant left frontal focus on ESI; the cross-hair/ cross marks the point of maximal activation/ electrical activity. The lower two panels show the amount of movement in the concordant fMRI run as recorded by the camera before (PMC) and after (RP) prospective motion correction.
