# Supplementary material for: Camera-based Prospective Motion Correction in Paediatric Epilepsy Patients Enables EEG-fMRI Localization Even in High-motion States
Source: Brain Topogr. 2023 Mar 20;36(3):319–37. doi: 10.1007/s10548-023-00945-0 (PMC10164016; doi:10.1007/s10548-023-00945-0)

**Supplementary figure 2** – *Comparison of interictal epileptiform discharges (IEDs) in outside and inside scanner EEGs*


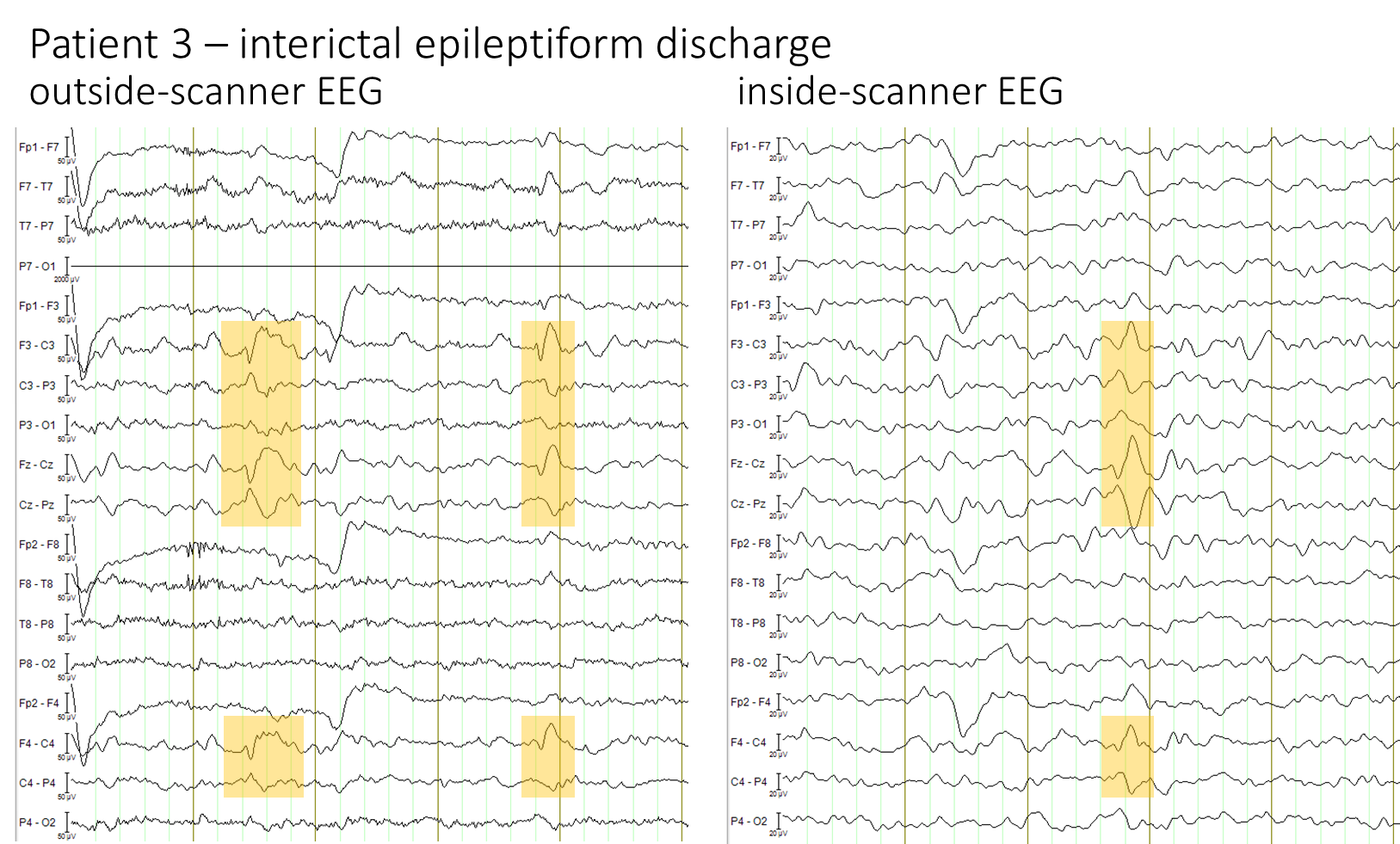


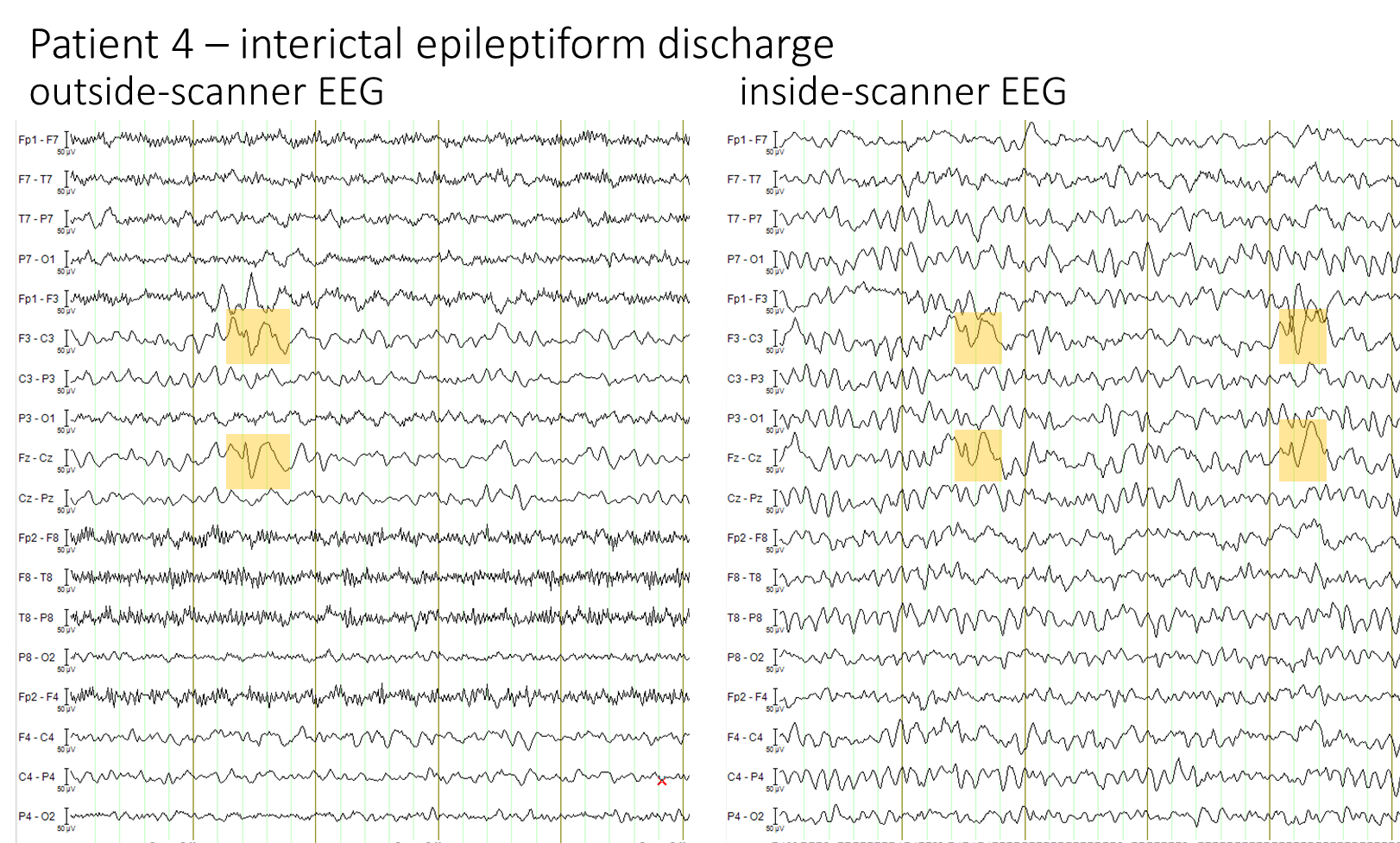


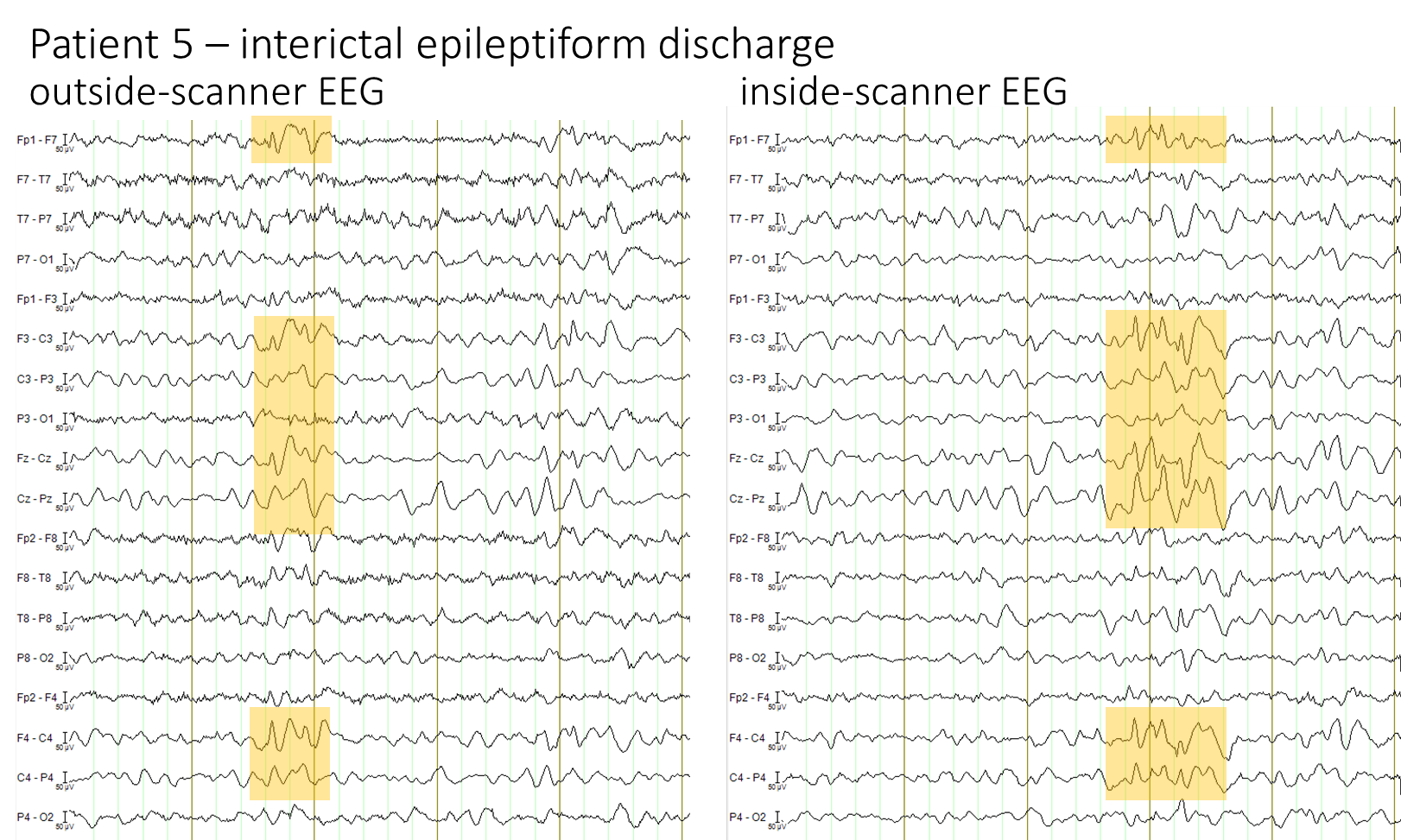


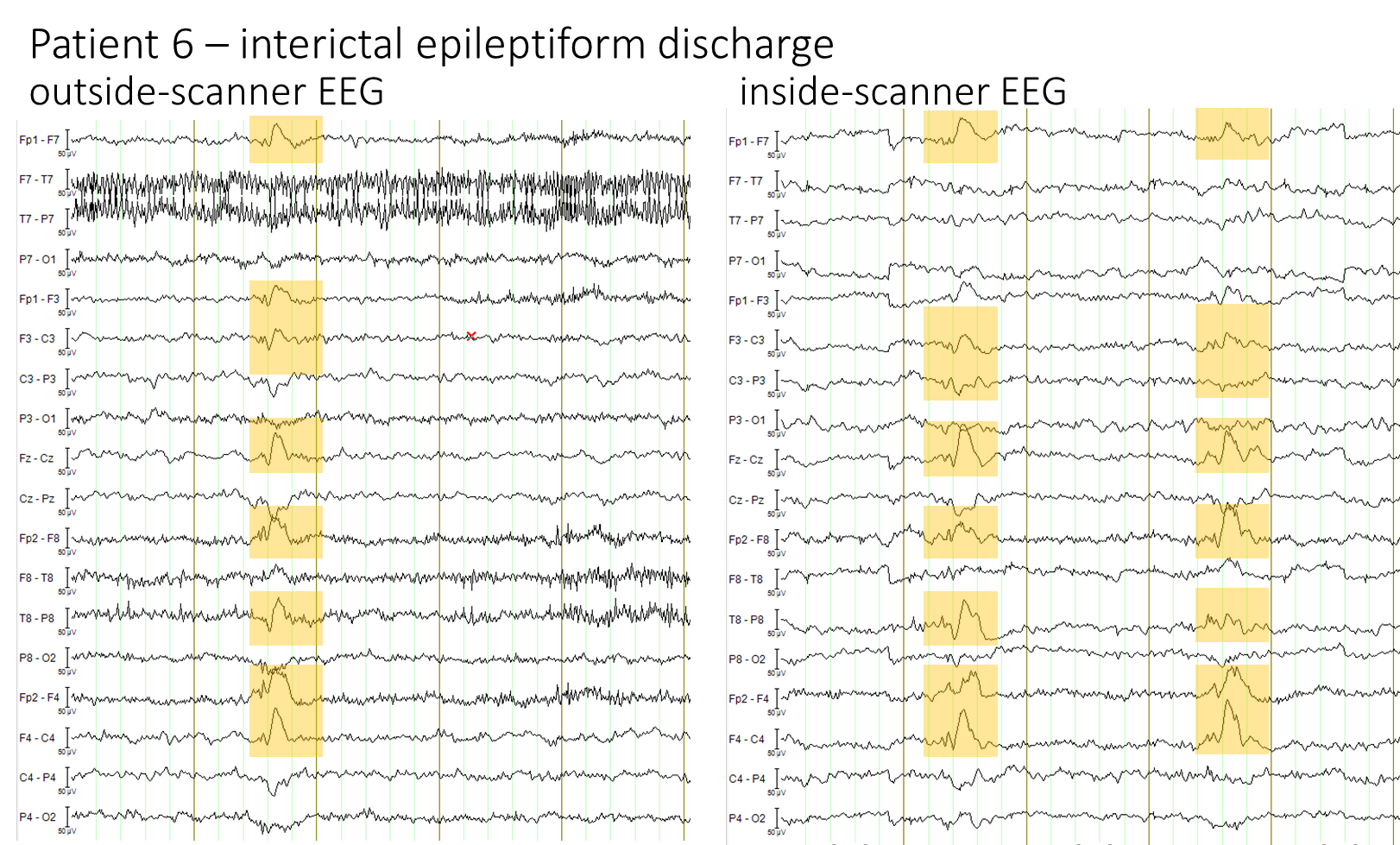


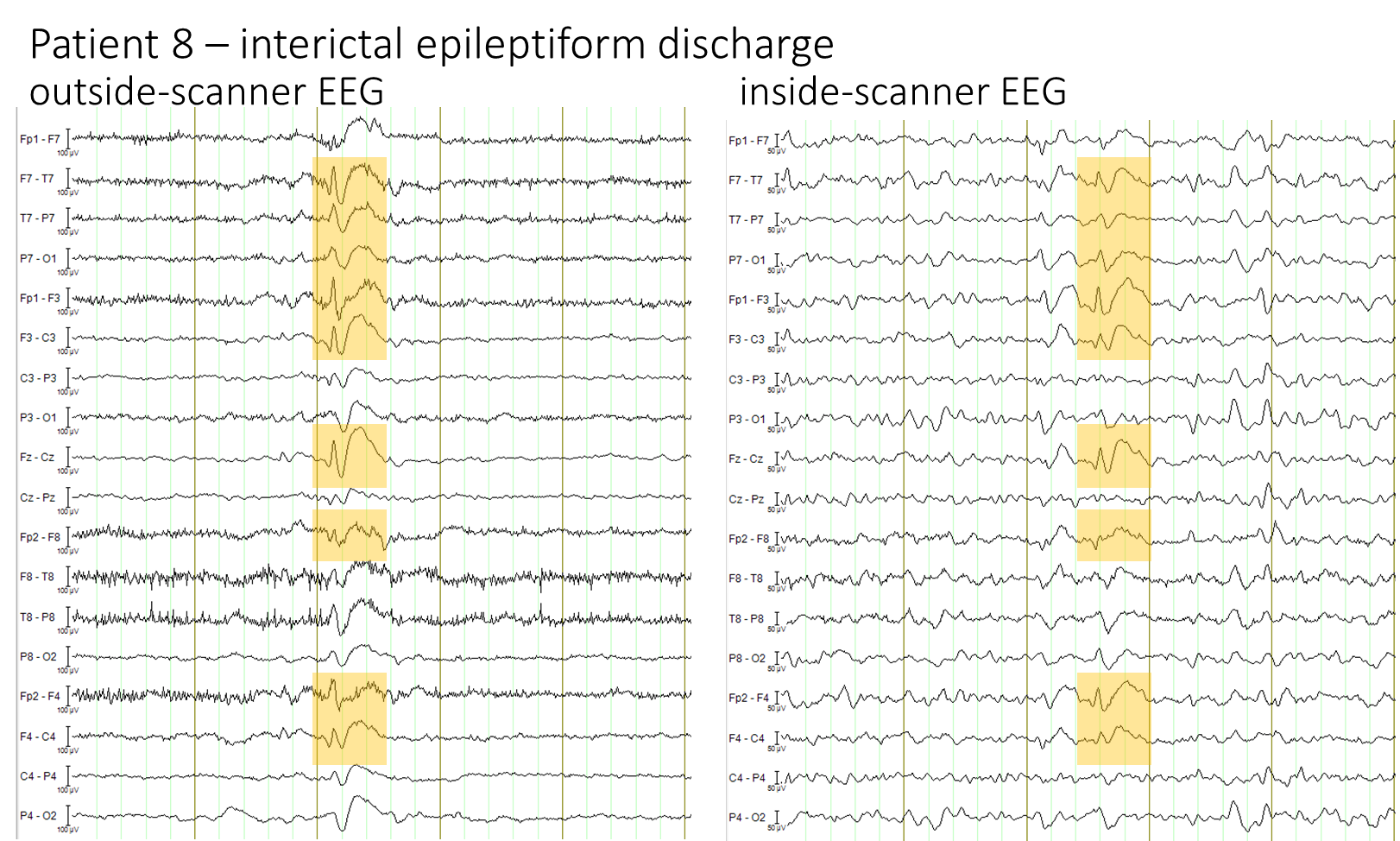


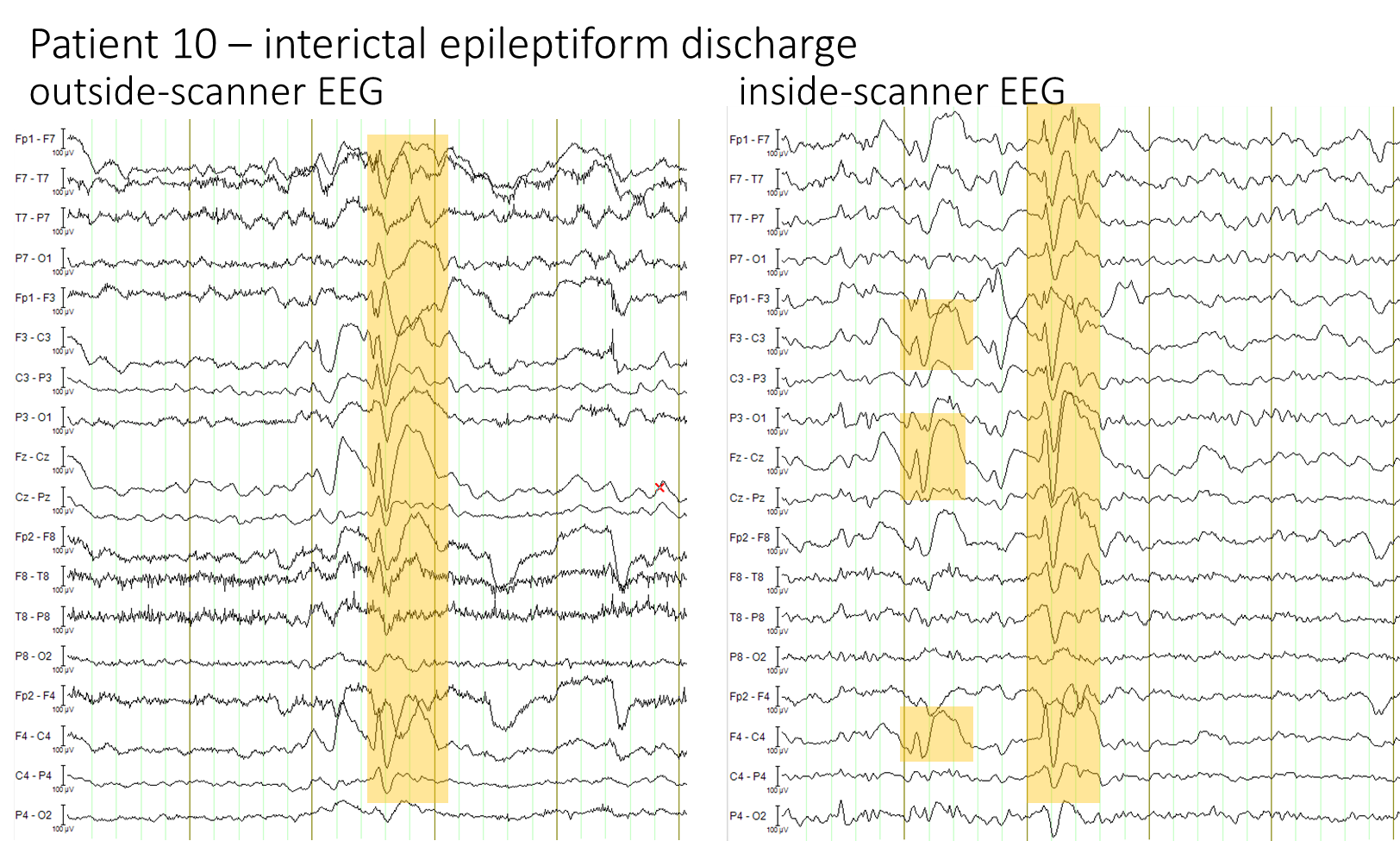

Supplement: Supplementary file 2 — Supplementary Material 2 [file 10548_2023_945_MOESM2_ESM.docx]
